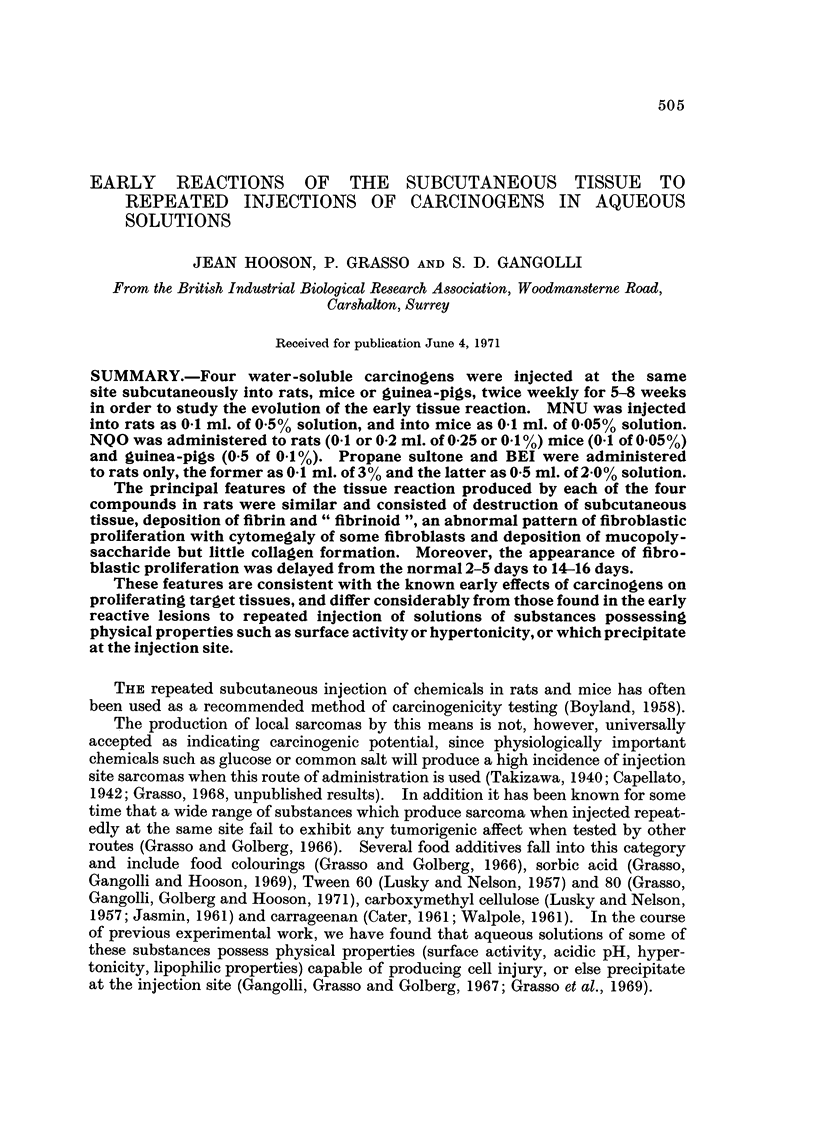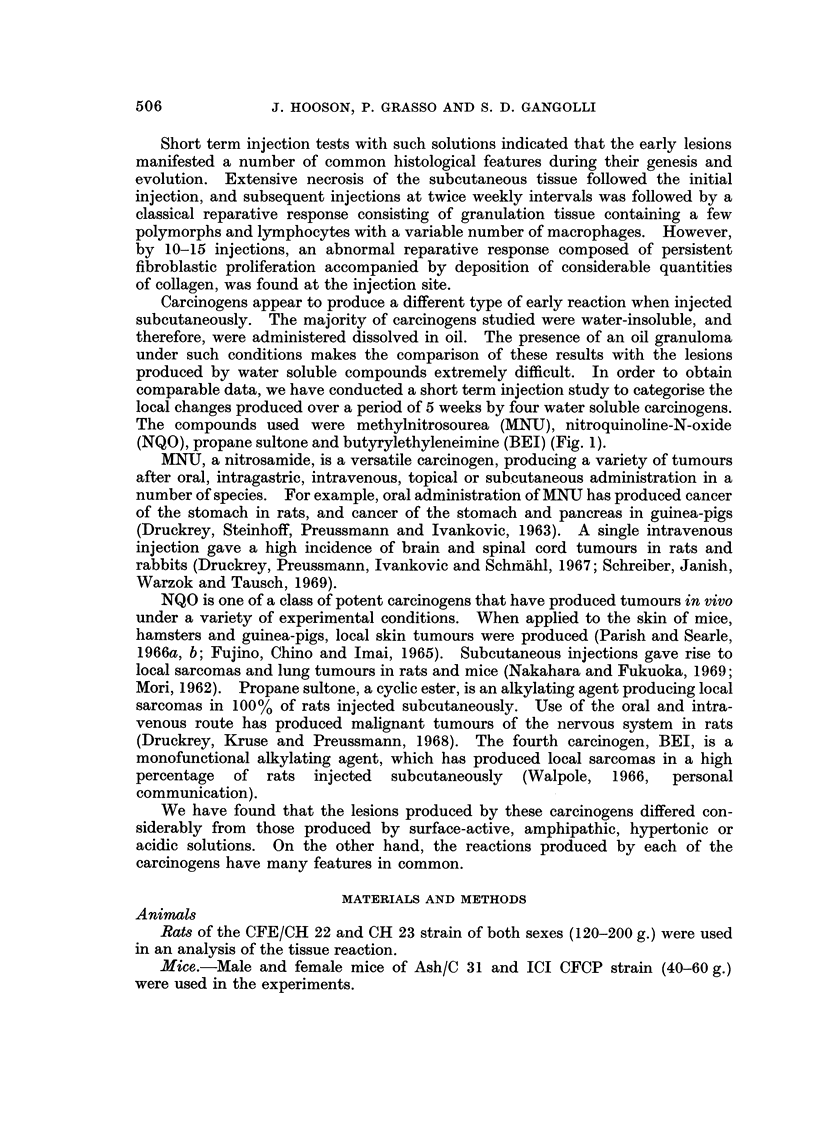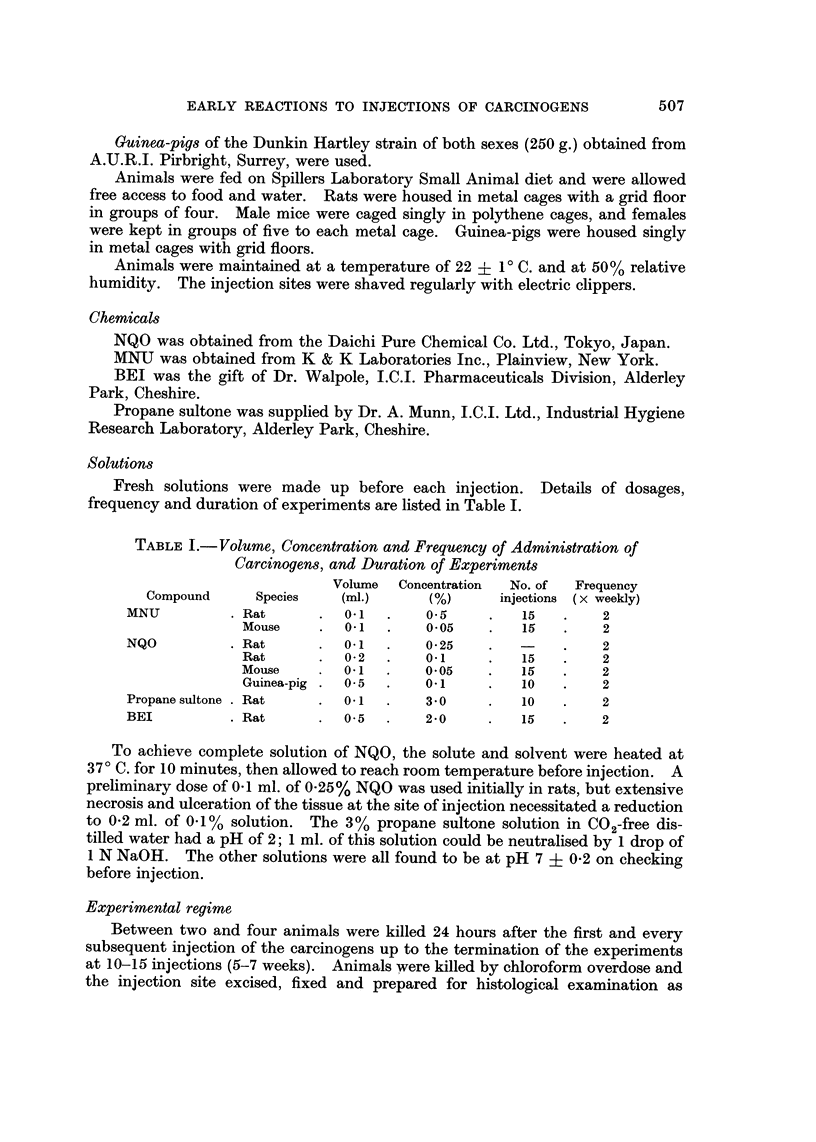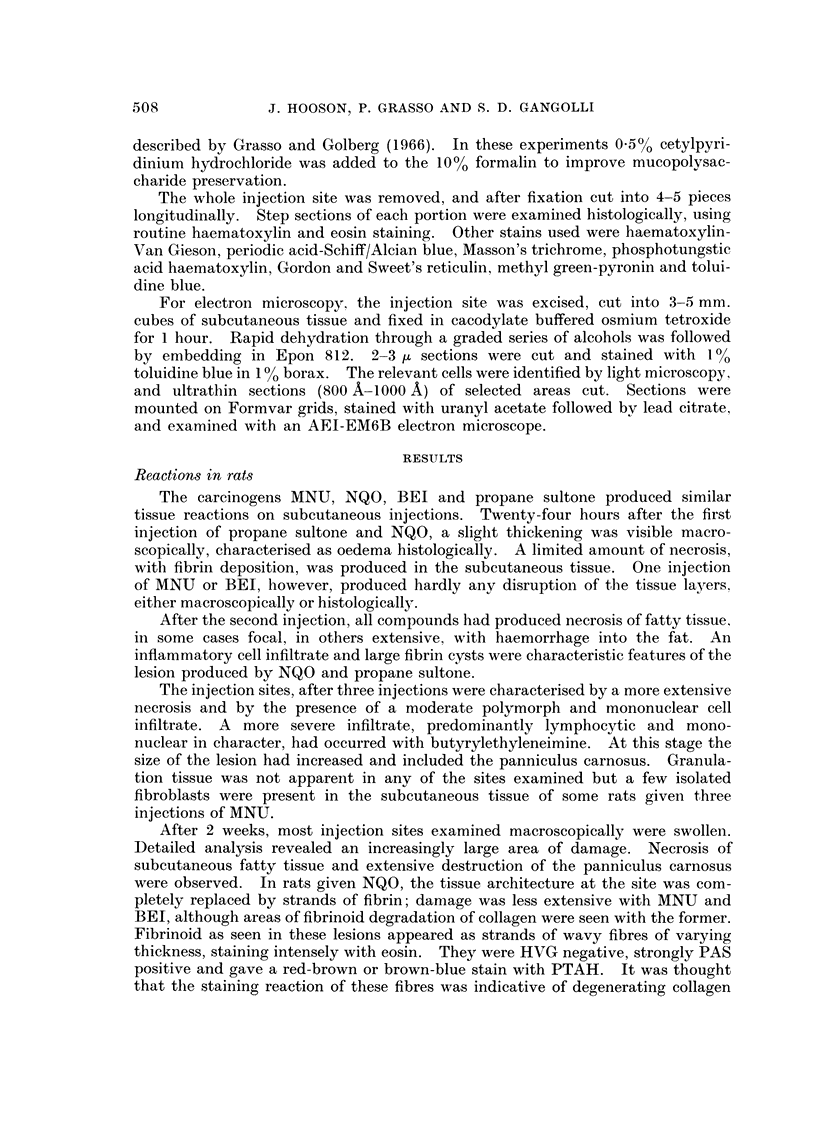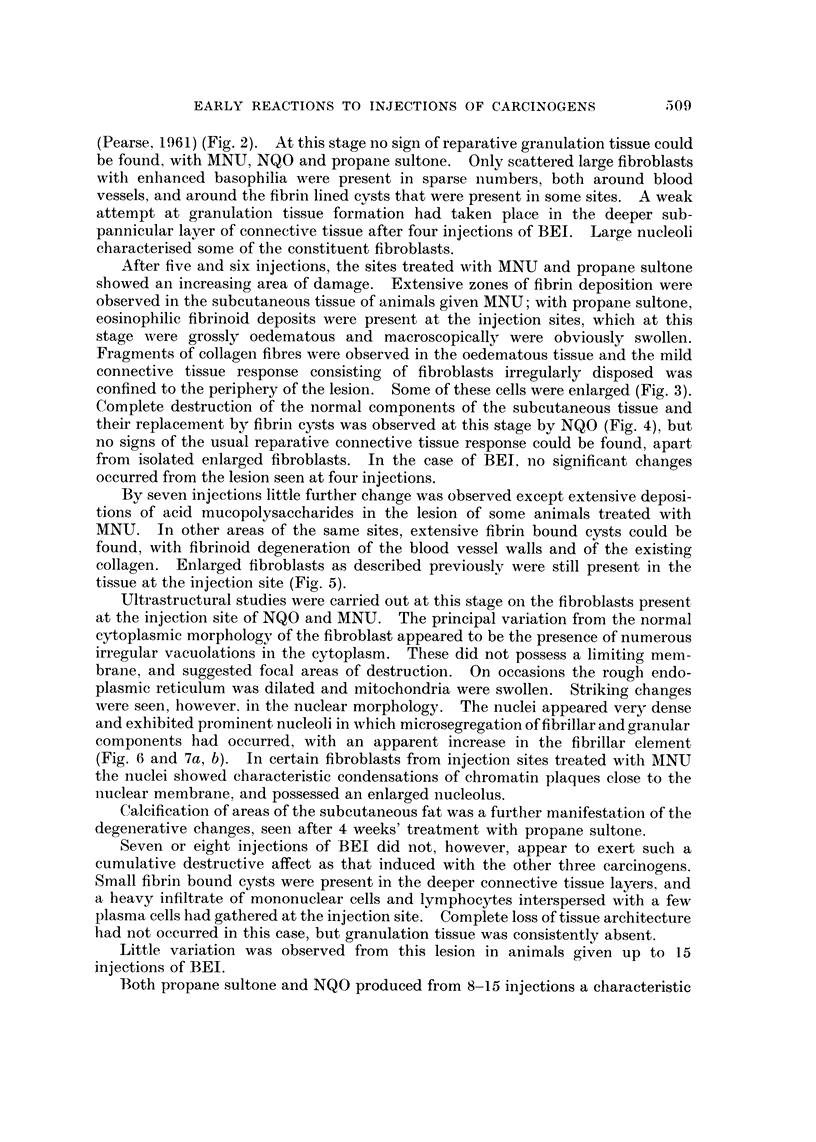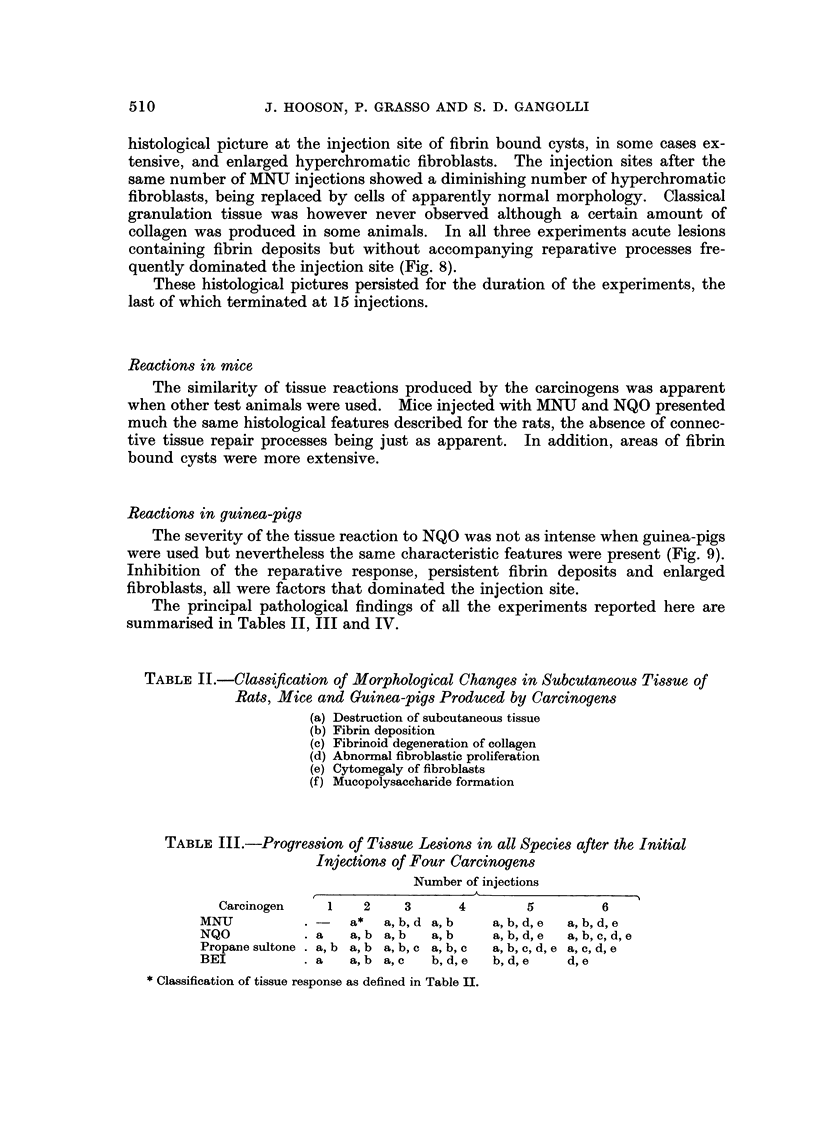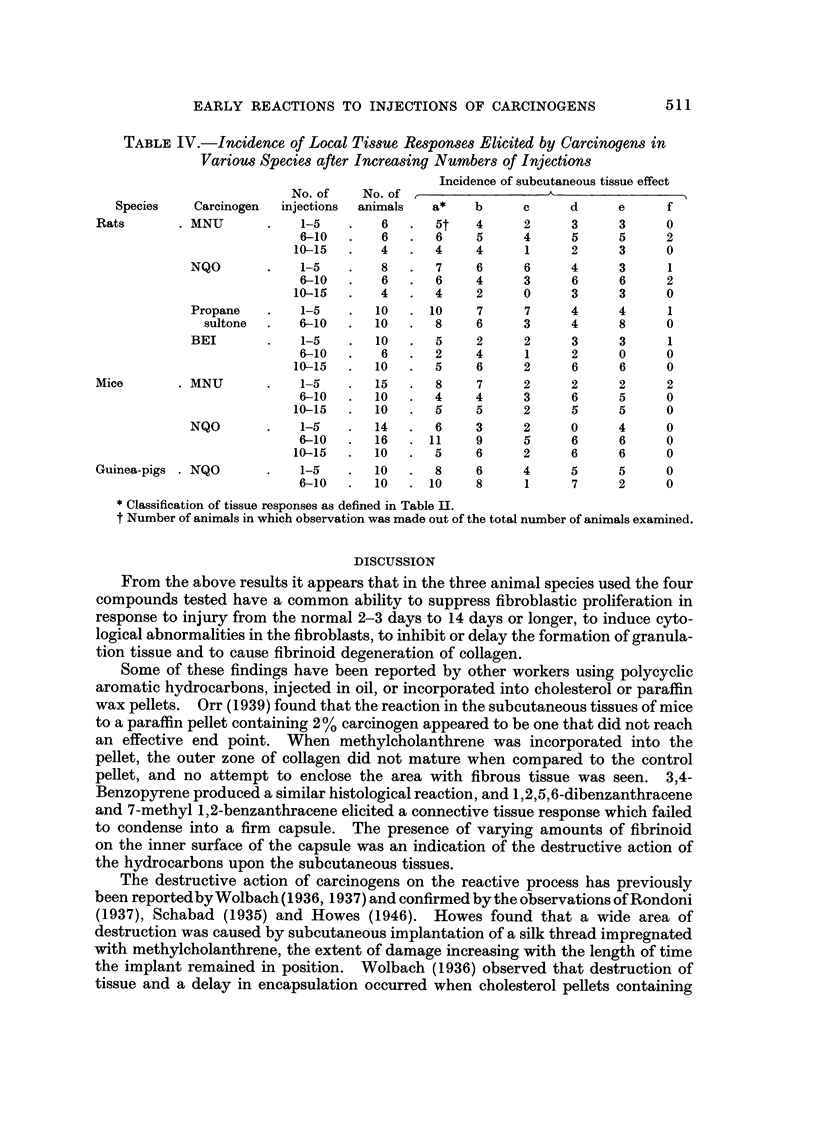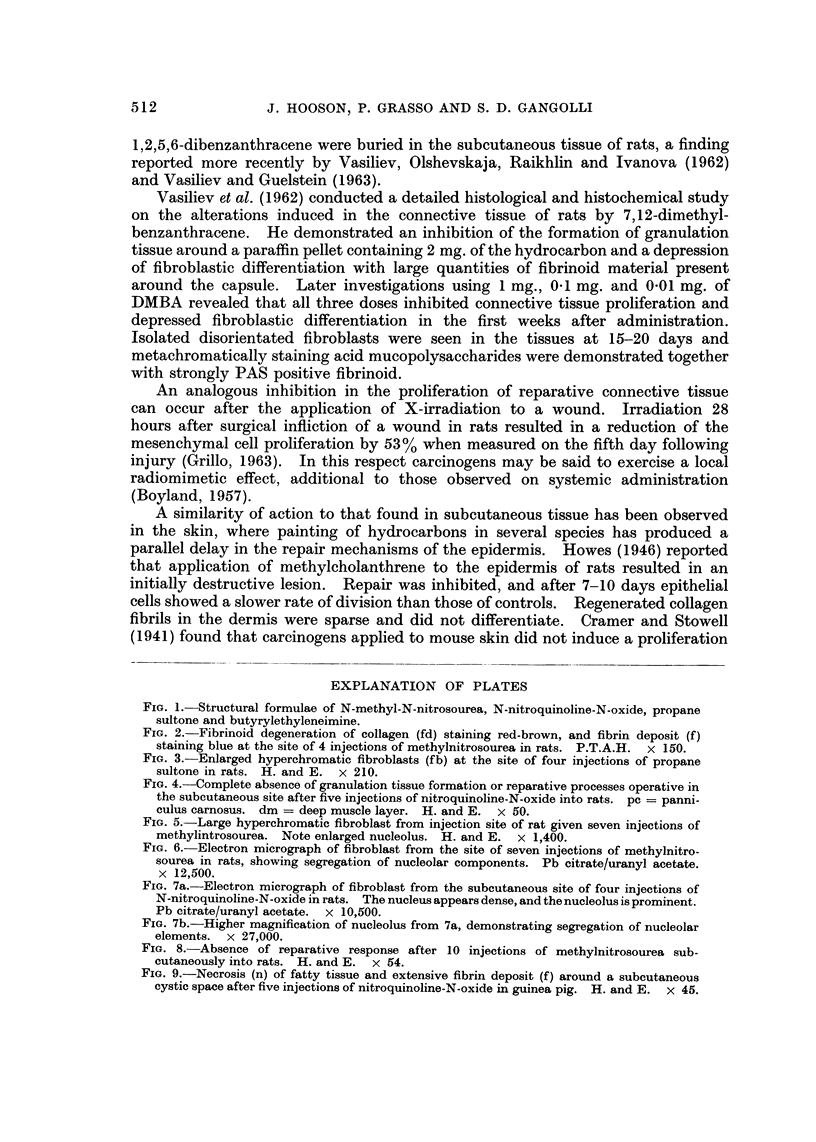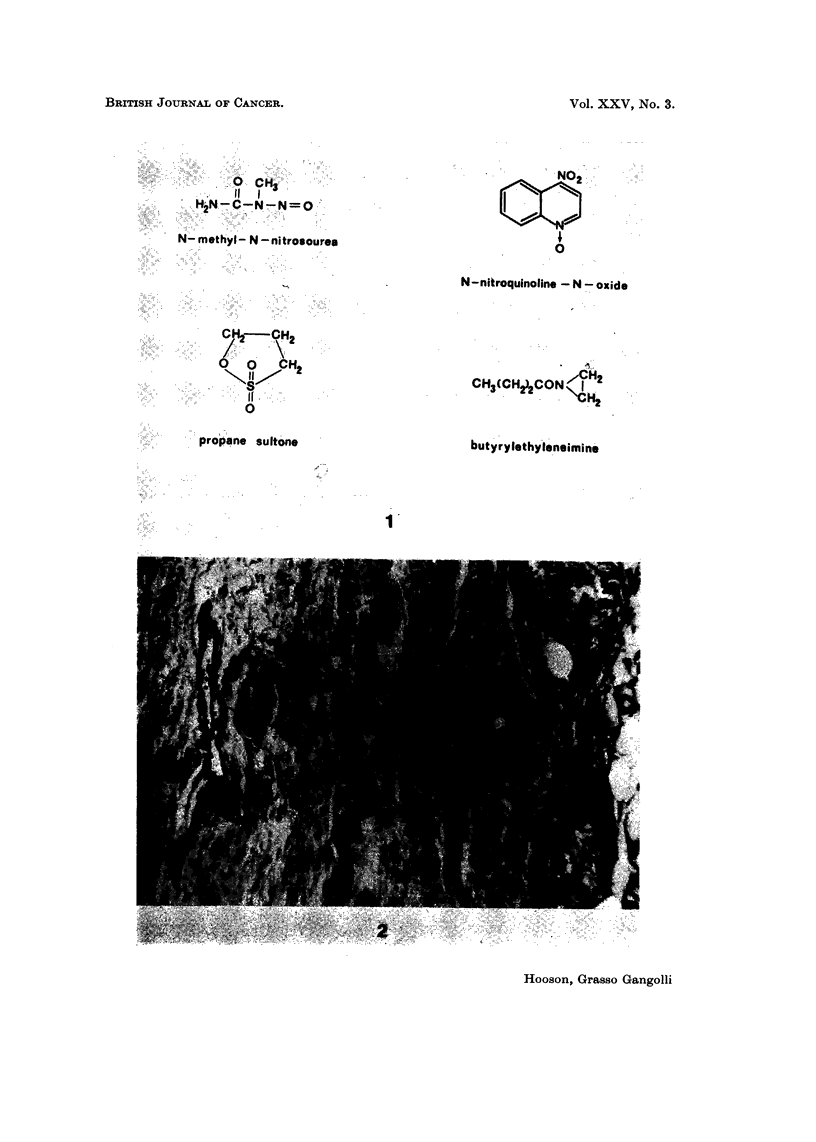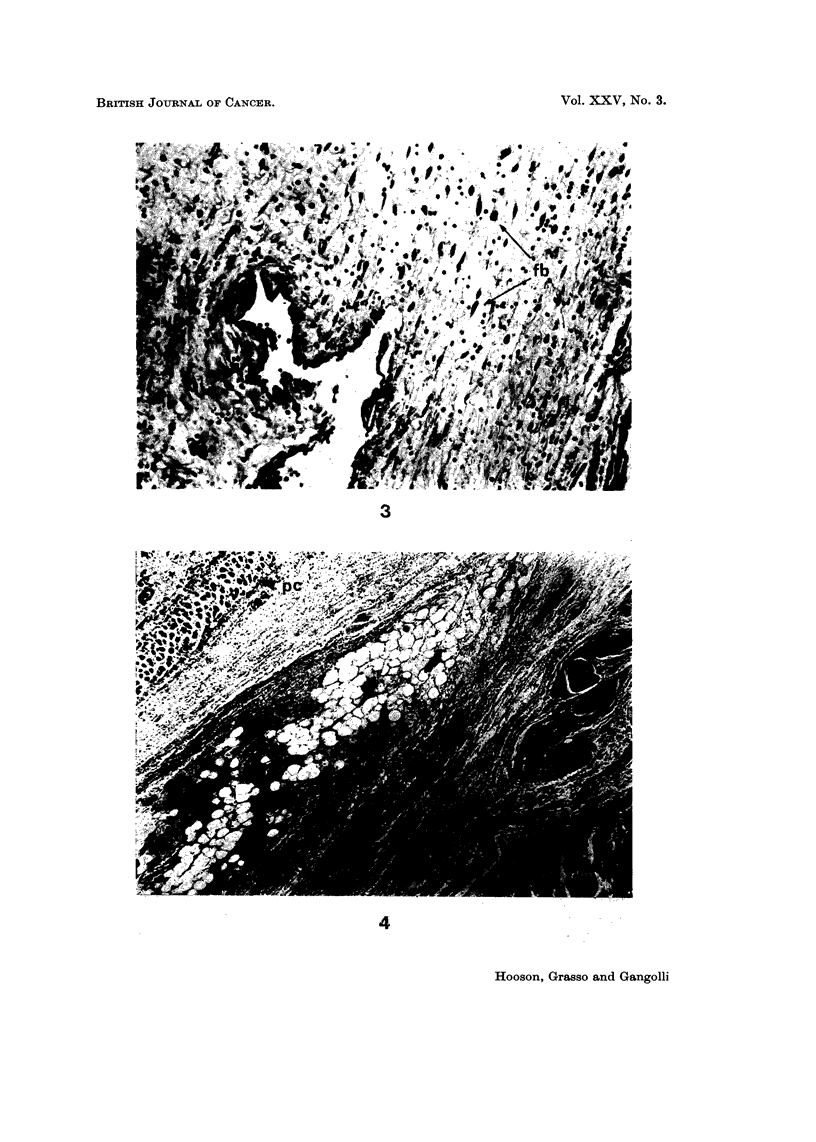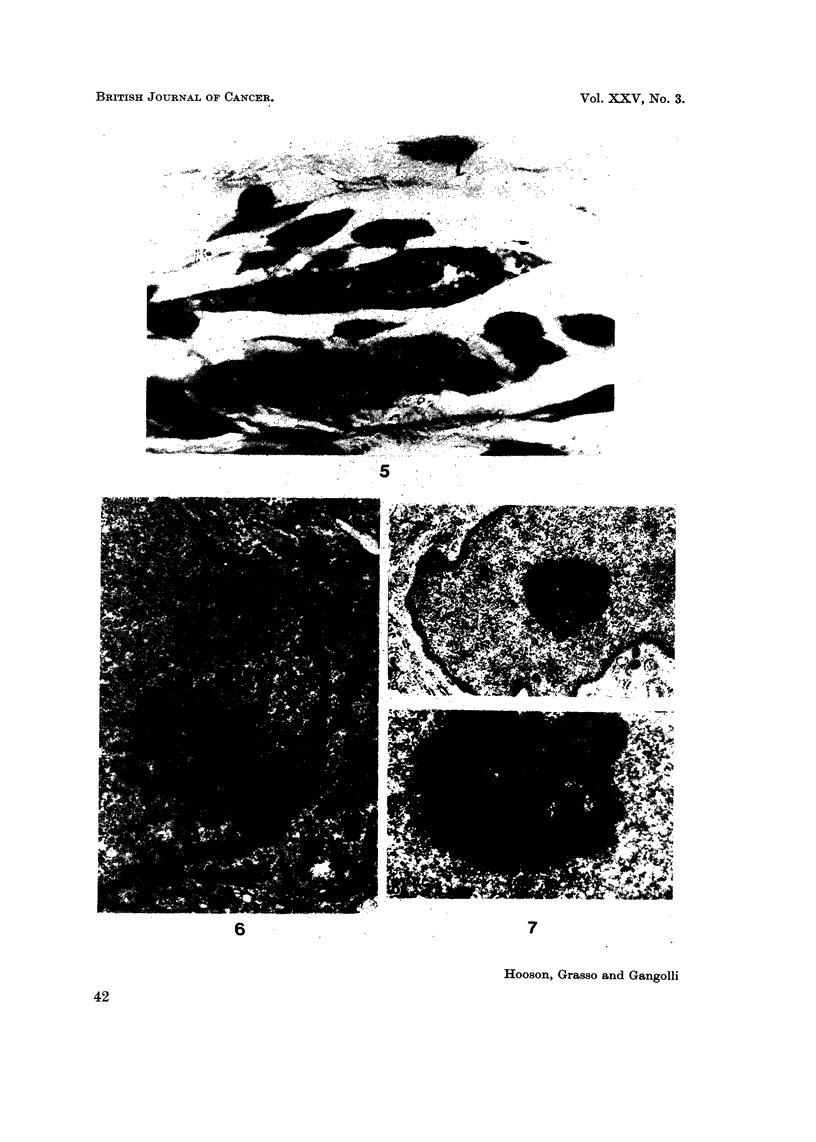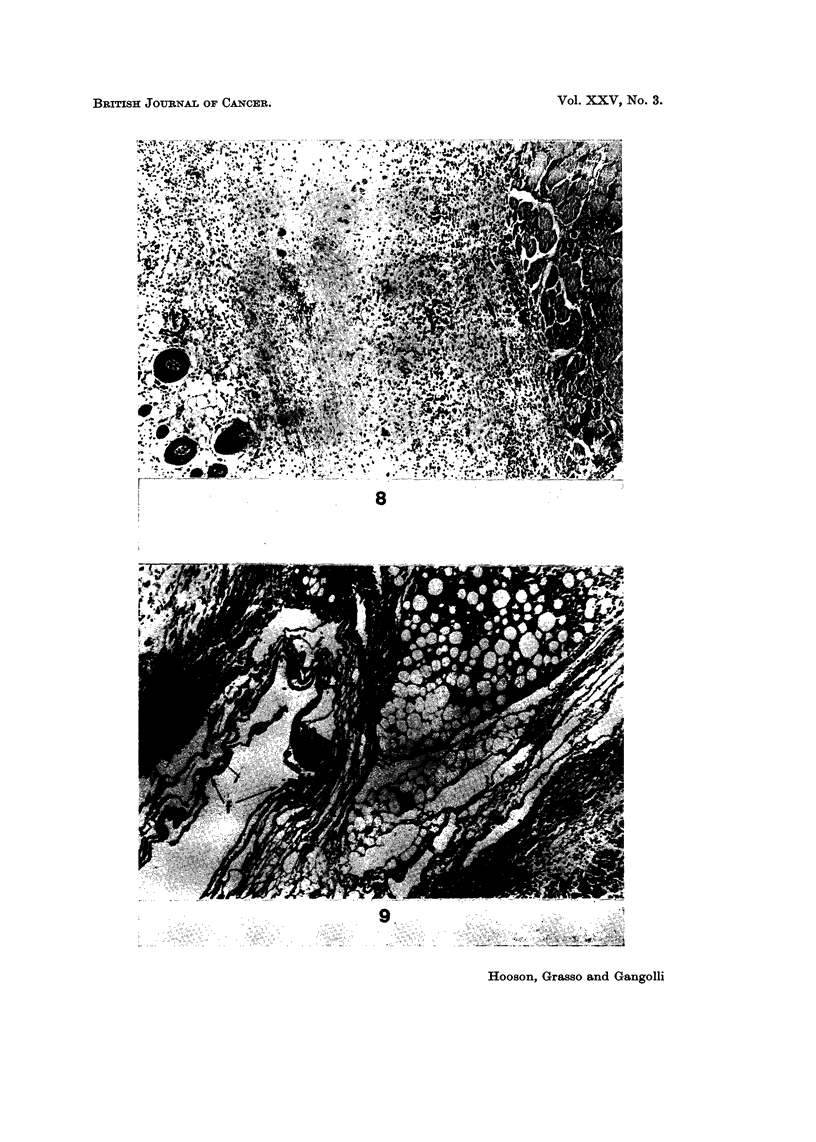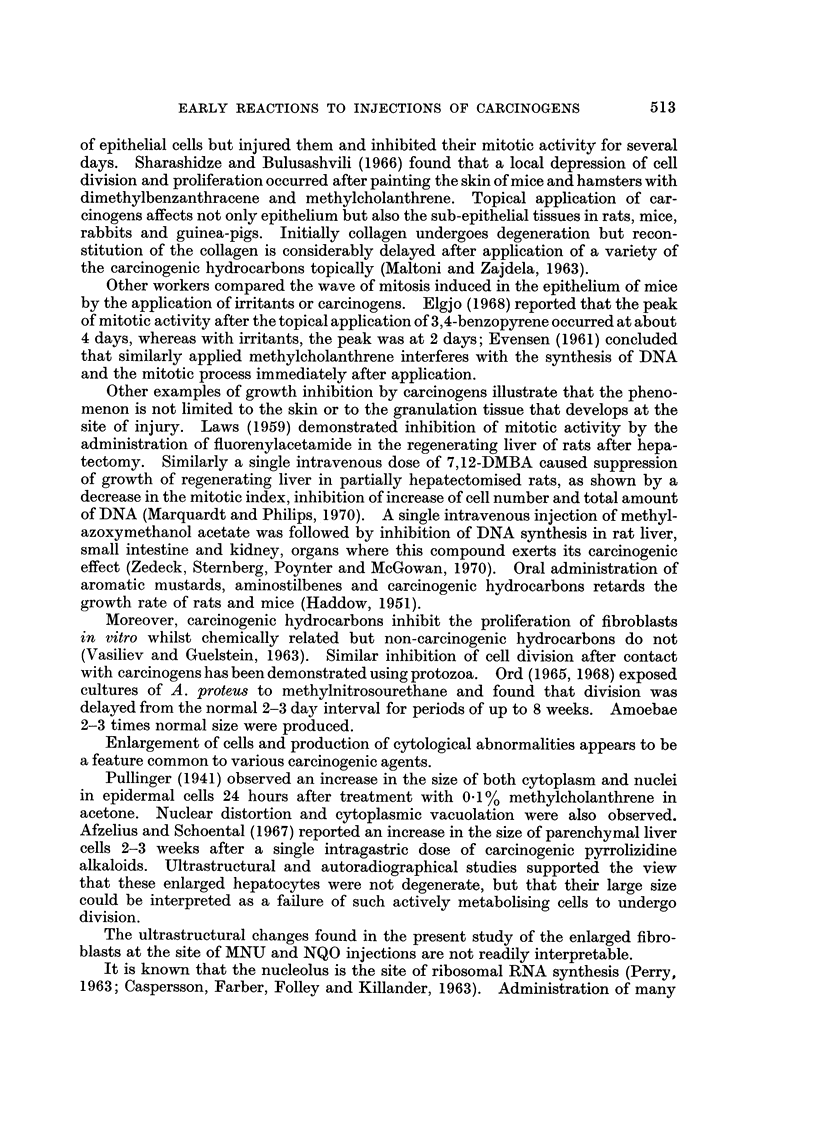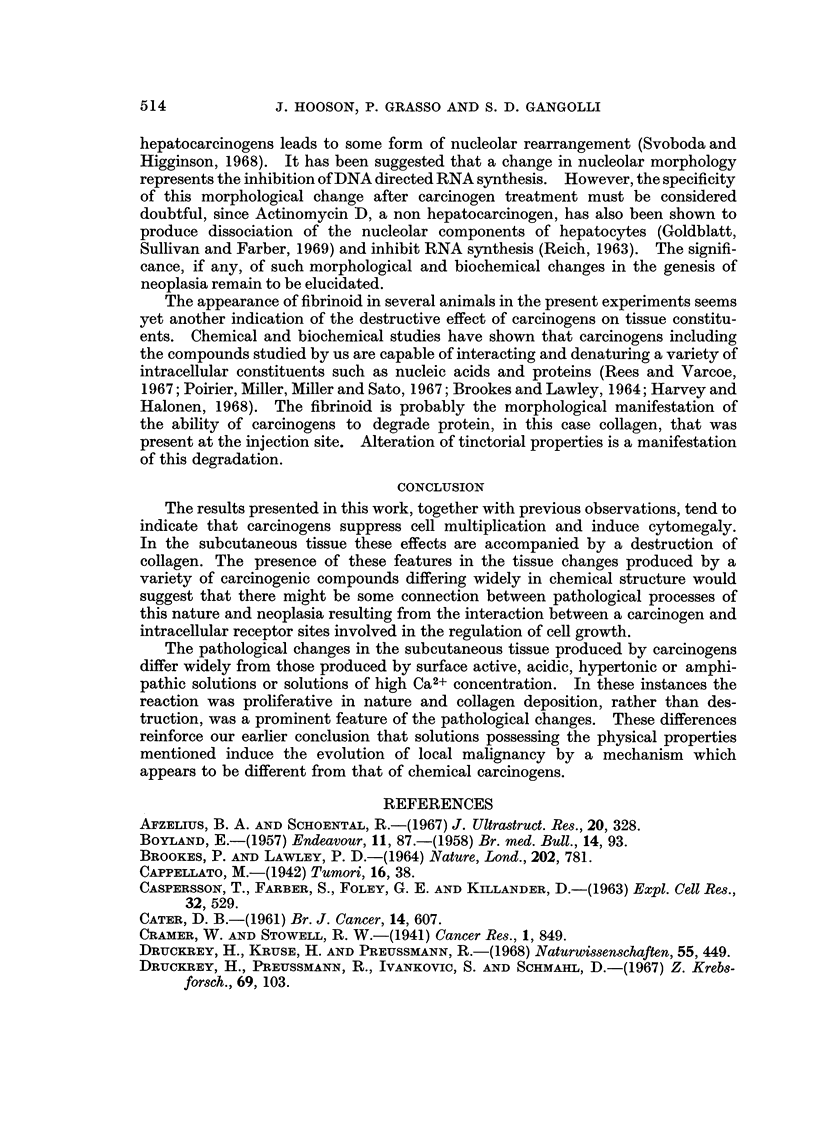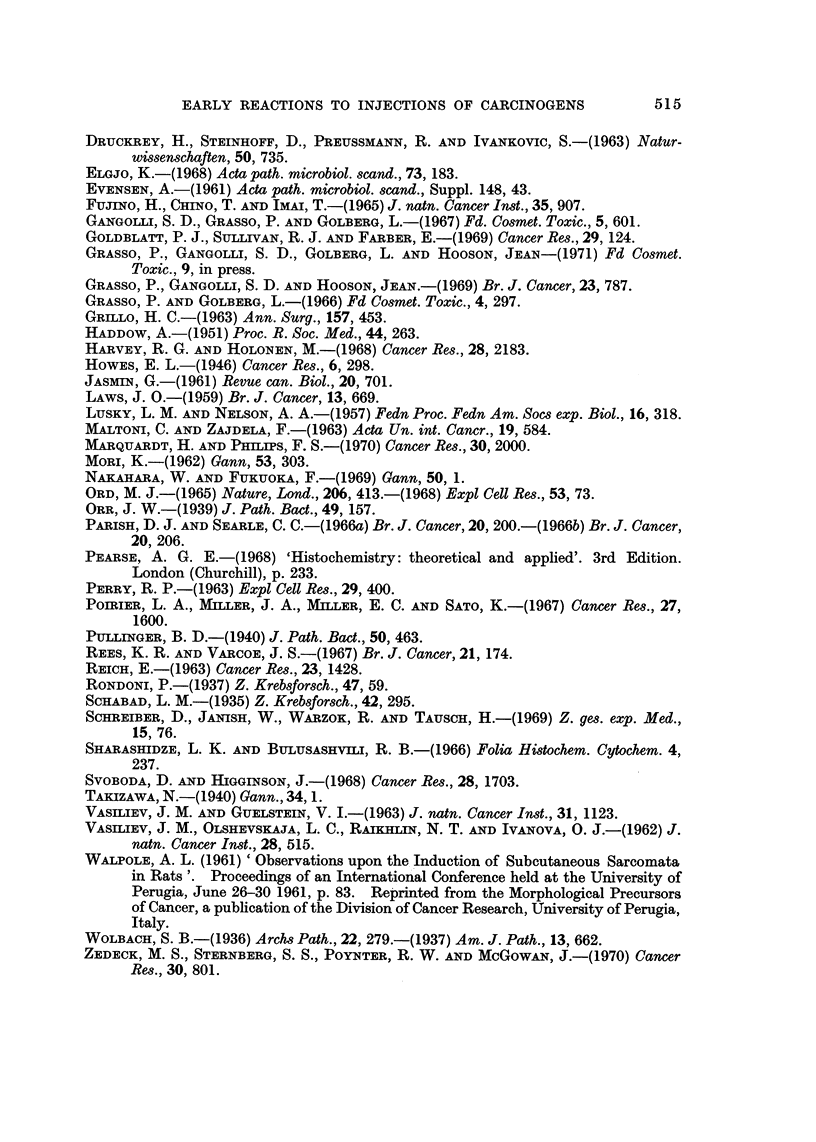# Early Reactions of the Subcutaneous Tissue to Repeated Injections of Carcinogens in Aqueous Solutions

**DOI:** 10.1038/bjc.1971.64

**Published:** 1971-09

**Authors:** Jean Hooson, P. Grasso, S. D. Gangolli

## Abstract

**Images:**


### EARLY REACTIONS OF THE SUBCUTANEOUS TISSUE TO REPEATED INJECTIONS OF CARCINOGENS IN AQUEOUS SOLUTIONS

JEAN HOOSON, P. GRASSO AND S. D. GANGOLLI

*From the British Industrial Biological Research Association, Woodmansterne Road, Carshalton, Surrey*

Received for publication June 4, 1971

**SUMMARY.—Four water-soluble carcinogens were injected at the same site subcutaneously into rats, mice or guinea-pigs, twice weekly for 5–8 weeks in order to study the evolution of the early tissue reaction. MNU was injected into rats as 0·1 ml. of 0·5% solution, and into mice as 0·1 ml. of 0·05% solution. NQO was administered to rats (0·1 or 0·2 ml. of 0·25 or 0·1%) mice (0·1 of 0·05%) and guinea-pigs (0·5 of 0·1%). Propane sultone and BEI were administered to rats only, the former as 0·1 ml. of 3% and the latter as 0·5 ml. of 2·0% solution.**

**The principal features of the tissue reaction produced by each of the four compounds in rats were similar and consisted of destruction of subcutaneous tissue, deposition of fibrin and " fibrinoid ", an abnormal pattern of fibroblastic proliferation with cytomegaly of some fibroblasts and deposition of mucopolysaccharide but little collagen formation. Moreover, the appearance of fibroblastic proliferation was delayed from the normal 2–5 days to 14–16 days.**

**These features are consistent with the known early effects of carcinogens on proliferating target tissues, and differ considerably from those found in the early reactive lesions to repeated injection of solutions of substances possessing physical properties such as surface activity or hypertonicity, or which precipitate at the injection site.**

THE repeated subcutaneous injection of chemicals in rats and mice has often been used as a recommended method of carcinogenicity testing (Boyland, 1958).

The production of local sarcomas by this means is not, however, universally accepted as indicating carcinogenic potential, since physiologically important chemicals such as glucose or common salt will produce a high incidence of injection site sarcomas when this route of administration is used (Takizawa, 1940; Capellato, 1942; Grasso, 1968, unpublished results). In addition it has been known for some time that a wide range of substances which produce sarcoma when injected repeatedly at the same site fail to exhibit any tumorigenic affect when tested by other routes (Grasso and Golberg, 1966). Several food additives fall into this category and include food colourings (Grasso and Golberg, 1966), sorbic acid (Grasso, Gangolli and Hooson, 1969), Tween 60 (Lusky and Nelson, 1957) and 80 (Grasso, Gangolli, Golberg and Hooson, 1971), carboxymethyl cellulose (Lusky and Nelson, 1957; Jasmin, 1961) and carrageenan (Cater, 1961; Walpole, 1961). In the course of previous experimental work, we have found that aqueous solutions of some of these substances possess physical properties (surface activity, acidic pH, hypertonicity, lipophilic properties) capable of producing cell injury, or else precipitate at the injection site (Gangolli, Grasso and Golberg, 1967; Grasso *et al.*, 1969).

Short term injection tests with such solutions indicated that the early lesions manifested a number of common histological features during their genesis and evolution. Extensive necrosis of the subcutaneous tissue followed the initial injection, and subsequent injections at twice weekly intervals was followed by a classical reparative response consisting of granulation tissue containing a few polymorphs and lymphocytes with a variable number of macrophages. However, by 10–15 injections, an abnormal reparative response composed of persistent fibroblastic proliferation accompanied by deposition of considerable quantities of collagen, was found at the injection site.

Carcinogens appear to produce a different type of early reaction when injected subcutaneously. The majority of carcinogens studied were water-insoluble, and therefore, were administered dissolved in oil. The presence of an oil granuloma under such conditions makes the comparison of these results with the lesions produced by water soluble compounds extremely difficult. In order to obtain comparable data, we have conducted a short term injection study to categorise the local changes produced over a period of 5 weeks by four water soluble carcinogens. The compounds used were methylnitrosourea (MNU), nitroquinoline-N-oxide (NQO), propane sultone and butyrylethyleneimine (BEI) (Fig. 1).

MNU, a nitrosamide, is a versatile carcinogen, producing a variety of tumours after oral, intragastric, intravenous, topical or subcutaneous administration in a number of species. For example, oral administration of MNU has produced cancer of the stomach in rats, and cancer of the stomach and pancreas in guinea-pigs (Druckrey, Steinhoff, Preussmann and Ivankovic, 1963). A single intravenous injection gave a high incidence of brain and spinal cord tumours in rats and rabbits (Druckrey, Preussmann, Ivankovic and Schmähl, 1967; Schreiber, Janish, Warzok and Tausch, 1969).

NQO is one of a class of potent carcinogens that have produced tumours *in vivo* under a variety of experimental conditions. When applied to the skin of mice, hamsters and guinea-pigs, local skin tumours were produced (Parish and Searle, 1966*a*, *b*; Fujino, Chino and Imai, 1965). Subcutaneous injections gave rise to local sarcomas and lung tumours in rats and mice (Nakahara and Fukuoka, 1969; Mori, 1962). Propane sultone, a cyclic ester, is an alkylating agent producing local sarcomas in 100% of rats injected subcutaneously. Use of the oral and intravenous route has produced malignant tumours of the nervous system in rats (Druckrey, Kruse and Preussmann, 1968). The fourth carcinogen, BEI, is a monofunctional alkylating agent, which has produced local sarcomas in a high percentage of rats injected subcutaneously (Walpole, 1966, personal communication).

We have found that the lesions produced by these carcinogens differed considerably from those produced by surface-active, amphipathic, hypertonic or acidic solutions. On the other hand, the reactions produced by each of the carcinogens have many features in common.

### MATERIALS AND METHODS

### *Animals*

*Rats* of the CFE/CH 22 and CH 23 strain of both sexes (120–200 g.) were used in an analysis of the tissue reaction.

*Mice.*—Male and female mice of Ash/C 31 and ICI CFCP strain (40–60 g.) were used in the experiments.

*Guinea-pigs* of the Dunkin Hartley strain of both sexes (250 g.) obtained from A.U.R.I. Pirbright, Surrey, were used.

Animals were fed on Spillers Laboratory Small Animal diet and were allowed free access to food and water. Rats were housed in metal cages with a grid floor in groups of four. Male mice were caged singly in polythene cages, and females were kept in groups of five to each metal cage. Guinea-pigs were housed singly in metal cages with grid floors.

Animals were maintained at a temperature of 22 ± 1° C. and at 50% relative humidity. The injection sites were shaved regularly with electric clippers.

*Chemicals*

NQO was obtained from the Daichi Pure Chemical Co. Ltd., Tokyo, Japan.

MNU was obtained from K & K Laboratories Inc., Plainview, New York.

BEI was the gift of Dr. Walpole, I.C.I. Pharmaceuticals Division, Alderley Park, Cheshire.

Propane sultone was supplied by Dr. A. Munn, I.C.I. Ltd., Industrial Hygiene Research Laboratory, Alderley Park, Cheshire.

*Solutions*

Fresh solutions were made up before each injection. Details of dosages, frequency and duration of experiments are listed in Table I.

TABLE I.—*Volume, Concentration and Frequency of Administration of Carcinogens, and Duration of Experiments*

| Compound | Species | Volume (ml.) | Concentration (%) | No. of injections | Frequency (× weekly) |
|---|---|---|---|---|---|
| MNU | Rat | 0·1 | 0·5 | 15 | 2 |
| | Mouse | 0·1 | 0·05 | 15 | 2 |
| NQO | Rat | 0·1 | 0·25 | — | 2 |
| | Rat | 0·2 | 0·1 | 15 | 2 |
| | Mouse | 0·1 | 0·05 | 15 | 2 |
| | Guinea-pig | 0·5 | 0·1 | 10 | 2 |
| Propane sultone | Rat | 0·1 | 3·0 | 10 | 2 |
| BEI | Rat | 0·5 | 2·0 | 15 | 2 |

To achieve complete solution of NQO, the solute and solvent were heated at 37° C. for 10 minutes, then allowed to reach room temperature before injection. A preliminary dose of 0·1 ml. of 0·25% NQO was used initially in rats, but extensive necrosis and ulceration of the tissue at the site of injection necessitated a reduction to 0·2 ml. of 0·1% solution. The 3% propane sultone solution in $CO_2$-free distilled water had a pH of 2; 1 ml. of this solution could be neutralised by 1 drop of 1 N NaOH. The other solutions were all found to be at pH 7 ± 0·2 on checking before injection.

*Experimental regime*

Between two and four animals were killed 24 hours after the first and every subsequent injection of the carcinogens up to the termination of the experiments at 10–15 injections (5–7 weeks). Animals were killed by chloroform overdose and the injection site excised, fixed and prepared for histological examination as described by Grasso and Golberg (1966). In these experiments 0·5% cetylpyridinium hydrochloride was added to the 10% formalin to improve mucopolysaccharide preservation.

The whole injection site was removed, and after fixation cut into 4–5 pieces longitudinally. Step sections of each portion were examined histologically, using routine haematoxylin and eosin staining. Other stains used were haematoxylin-Van Gieson, periodic acid-Schiff/Alcian blue, Masson's trichrome, phosphotungstic acid haematoxylin, Gordon and Sweet's reticulin, methyl green-pyronin and toluidine blue.

For electron microscopy, the injection site was excised, cut into 3–5 mm. cubes of subcutaneous tissue and fixed in cacodylate buffered osmium tetroxide for 1 hour. Rapid dehydration through a graded series of alcohols was followed by embedding in Epon 812. 2–3 $\mu$ sections were cut and stained with 1% toluidine blue in 1% borax. The relevant cells were identified by light microscopy, and ultrathin sections (800 Å–1000 Å) of selected areas cut. Sections were mounted on Formvar grids, stained with uranyl acetate followed by lead citrate, and examined with an AEI-EM6B electron microscope.

### RESULTS

### *Reactions in rats*

The carcinogens MNU, NQO, BEI and propane sultone produced similar tissue reactions on subcutaneous injections. Twenty-four hours after the first injection of propane sultone and NQO, a slight thickening was visible macroscopically, characterised as oedema histologically. A limited amount of necrosis, with fibrin deposition, was produced in the subcutaneous tissue. One injection of MNU or BEI, however, produced hardly any disruption of the tissue layers, either macroscopically or histologically.

After the second injection, all compounds had produced necrosis of fatty tissue, in some cases focal, in others extensive, with haemorrhage into the fat. An inflammatory cell infiltrate and large fibrin cysts were characteristic features of the lesion produced by NQO and propane sultone.

The injection sites, after three injections were characterised by a more extensive necrosis and by the presence of a moderate polymorph and mononuclear cell infiltrate. A more severe infiltrate, predominantly lymphocytic and mononuclear in character, had occurred with butyrylethyleneimine. At this stage the size of the lesion had increased and included the panniculus carnosus. Granulation tissue was not apparent in any of the sites examined but a few isolated fibroblasts were present in the subcutaneous tissue of some rats given three injections of MNU.

After 2 weeks, most injection sites examined macroscopically were swollen. Detailed analysis revealed an increasingly large area of damage. Necrosis of subcutaneous fatty tissue and extensive destruction of the panniculus carnosus were observed. In rats given NQO, the tissue architecture at the site was completely replaced by strands of fibrin; damage was less extensive with MNU and BEI, although areas of fibrinoid degradation of collagen were seen with the former. Fibrinoid as seen in these lesions appeared as strands of wavy fibres of varying thickness, staining intensely with eosin. They were HVG negative, strongly PAS positive and gave a red-brown or brown-blue stain with PTAH. It was thought that the staining reaction of these fibres was indicative of degenerating collagen

(Pearse, 1961) (Fig. 2). At this stage no sign of reparative granulation tissue could be found, with MNU, NQO and propane sultone. Only scattered large fibroblasts with enhanced basophilia were present in sparse numbers, both around blood vessels, and around the fibrin lined cysts that were present in some sites. A weak attempt at granulation tissue formation had taken place in the deeper subpannicular layer of connective tissue after four injections of BEI. Large nucleoli characterised some of the constituent fibroblasts.

After five and six injections, the sites treated with MNU and propane sultone showed an increasing area of damage. Extensive zones of fibrin deposition were observed in the subcutaneous tissue of animals given MNU; with propane sultone, eosinophilic fibrinoid deposits were present at the injection sites, which at this stage were grossly oedematous and macroscopically were obviously swollen. Fragments of collagen fibres were observed in the oedematous tissue and the mild connective tissue response consisting of fibroblasts irregularly disposed was confined to the periphery of the lesion. Some of these cells were enlarged (Fig. 3). Complete destruction of the normal components of the subcutaneous tissue and their replacement by fibrin cysts was observed at this stage by NQO (Fig. 4), but no signs of the usual reparative connective tissue response could be found, apart from isolated enlarged fibroblasts. In the case of BEI, no significant changes occurred from the lesion seen at four injections.

By seven injections little further change was observed except extensive depositions of acid mucopolysaccharides in the lesion of some animals treated with MNU. In other areas of the same sites, extensive fibrin bound cysts could be found, with fibrinoid degeneration of the blood vessel walls and of the existing collagen. Enlarged fibroblasts as described previously were still present in the tissue at the injection site (Fig. 5).

Ultrastructural studies were carried out at this stage on the fibroblasts present at the injection site of NQO and MNU. The principal variation from the normal cytoplasmic morphology of the fibroblast appeared to be the presence of numerous irregular vacuolations in the cytoplasm. These did not possess a limiting membrane, and suggested focal areas of destruction. On occasions the rough endoplasmic reticulum was dilated and mitochondria were swollen. Striking changes were seen, however, in the nuclear morphology. The nuclei appeared very dense and exhibited prominent nucleoli in which microsegregation of fibrillar and granular components had occurred, with an apparent increase in the fibrillar element (Fig. 6 and 7*a*, *b*). In certain fibroblasts from injection sites treated with MNU the nuclei showed characteristic condensations of chromatin plaques close to the nuclear membrane, and possessed an enlarged nucleolus.

Calcification of areas of the subcutaneous fat was a further manifestation of the degenerative changes, seen after 4 weeks' treatment with propane sultone.

Seven or eight injections of BEI did not, however, appear to exert such a cumulative destructive affect as that induced with the other three carcinogens. Small fibrin bound cysts were present in the deeper connective tissue layers, and a heavy infiltrate of mononuclear cells and lymphocytes interspersed with a few plasma cells had gathered at the injection site. Complete loss of tissue architecture had not occurred in this case, but granulation tissue was consistently absent.

Little variation was observed from this lesion in animals given up to 15 injections of BEI.

Both propane sultone and NQO produced from 8–15 injections a characteristic histological picture at the injection site of fibrin bound cysts, in some cases extensive, and enlarged hyperchromatic fibroblasts. The injection sites after the same number of MNU injections showed a diminishing number of hyperchromatic fibroblasts, being replaced by cells of apparently normal morphology. Classical granulation tissue was however never observed although a certain amount of collagen was produced in some animals. In all three experiments acute lesions containing fibrin deposits but without accompanying reparative processes frequently dominated the injection site (Fig. 8).

These histological pictures persisted for the duration of the experiments, the last of which terminated at 15 injections.

### *Reactions in mice*

The similarity of tissue reactions produced by the carcinogens was apparent when other test animals were used. Mice injected with MNU and NQO presented much the same histological features described for the rats, the absence of connective tissue repair processes being just as apparent. In addition, areas of fibrin bound cysts were more extensive.

### *Reactions in guinea-pigs*

The severity of the tissue reaction to NQO was not as intense when guinea-pigs were used but nevertheless the same characteristic features were present (Fig. 9). Inhibition of the reparative response, persistent fibrin deposits and enlarged fibroblasts, all were factors that dominated the injection site.

The principal pathological findings of all the experiments reported here are summarised in Tables II, III and IV.

TABLE II.—*Classification of Morphological Changes in Subcutaneous Tissue of Rats, Mice and Guinea-pigs Produced by Carcinogens*

(a) Destruction of subcutaneous tissue
(b) Fibrin deposition
(c) Fibrinoid degeneration of collagen
(d) Abnormal fibroblastic proliferation
(e) Cytomegaly of fibroblasts
(f) Mucopolysaccharide formation

TABLE III.—*Progression of Tissue Lesions in all Species after the Initial Injections of Four Carcinogens*

| Carcinogen | Number of injections | | | | | |
|---|---|---|---|---|---|---|
| | 1 | 2 | 3 | 4 | 5 | 6 |
| MNU | — | a* | a, b, d | a, b | a, b, d, e | a, b, d, e |
| NQO | a | a, b | a, b | a, b | a, b, d, e | a, b, c, d, e |
| Propane sultone | a, b | a, b | a, b, c | a, b, c | a, b, c, d, e | a, c, d, e |
| BEI | a | a, b | a, c | b, d, e | b, d, e | d, e |

* Classification of tissue response as defined in Table II.

TABLE IV.—*Incidence of Local Tissue Responses Elicited by Carcinogens in Various Species after Increasing Numbers of Injections*

| Species | Carcinogen | No. of injections | No. of animals | Incidence of subcutaneous tissue effect a* | b | c | d | e | f |
|---|---|---|---|---|---|---|---|---|---|
| Rats | MNU | 1–5 | 6 | 5† | 4 | 2 | 3 | 3 | 0 |
| | | 6–10 | 6 | 6 | 5 | 4 | 5 | 5 | 2 |
| | | 10–15 | 4 | 4 | 4 | 1 | 2 | 3 | 0 |
| | NQO | 1–5 | 8 | 7 | 6 | 6 | 4 | 3 | 1 |
| | | 6–10 | 6 | 6 | 4 | 3 | 6 | 6 | 2 |
| | | 10–15 | 4 | 4 | 2 | 0 | 3 | 3 | 0 |
| | Propane | 1–5 | 10 | 10 | 7 | 7 | 4 | 4 | 1 |
| | sultone | 6–10 | 10 | 8 | 6 | 3 | 4 | 8 | 0 |
| | BEI | 1–5 | 10 | 5 | 2 | 2 | 3 | 3 | 1 |
| | | 6–10 | 6 | 2 | 4 | 1 | 2 | 0 | 0 |
| | | 10–15 | 10 | 5 | 6 | 2 | 6 | 6 | 0 |
| Mice | MNU | 1–5 | 15 | 8 | 7 | 2 | 2 | 2 | 2 |
| | | 6–10 | 10 | 4 | 4 | 3 | 6 | 5 | 0 |
| | | 10–15 | 10 | 5 | 5 | 2 | 5 | 5 | 0 |
| | NQO | 1–5 | 14 | 6 | 3 | 2 | 0 | 4 | 0 |
| | | 6–10 | 16 | 11 | 9 | 5 | 6 | 6 | 0 |
| | | 10–15 | 10 | 5 | 6 | 2 | 6 | 6 | 0 |
| Guinea-pigs | NQO | 1–5 | 10 | 8 | 6 | 4 | 5 | 5 | 0 |
| | | 6–10 | 10 | 10 | 8 | 1 | 7 | 2 | 0 |

* Classification of tissue responses as defined in Table II.
† Number of animals in which observation was made out of the total number of animals examined.

### DISCUSSION

From the above results it appears that in the three animal species used the four compounds tested have a common ability to suppress fibroblastic proliferation in response to injury from the normal 2–3 days to 14 days or longer, to induce cytological abnormalities in the fibroblasts, to inhibit or delay the formation of granulation tissue and to cause fibrinoid degeneration of collagen.

Some of these findings have been reported by other workers using polycyclic aromatic hydrocarbons, injected in oil, or incorporated into cholesterol or paraffin wax pellets. Orr (1939) found that the reaction in the subcutaneous tissues of mice to a paraffin pellet containing 2% carcinogen appeared to be one that did not reach an effective end point. When methylcholanthrene was incorporated into the pellet, the outer zone of collagen did not mature when compared to the control pellet, and no attempt to enclose the area with fibrous tissue was seen. 3,4-Benzopyrene produced a similar histological reaction, and 1,2,5,6-dibenzanthracene and 7-methyl 1,2-benzanthracene elicited a connective tissue response which failed to condense into a firm capsule. The presence of varying amounts of fibrinoid on the inner surface of the capsule was an indication of the destructive action of the hydrocarbons upon the subcutaneous tissues.

The destructive action of carcinogens on the reactive process has previously been reported by Wolbach (1936, 1937) and confirmed by the observations of Rondoni (1937), Schabad (1935) and Howes (1946). Howes found that a wide area of destruction was caused by subcutaneous implantation of a silk thread impregnated with methylcholanthrene, the extent of damage increasing with the length of time the implant remained in position. Wolbach (1936) observed that destruction of tissue and a delay in encapsulation occurred when cholesterol pellets containing

1,2,5,6-dibenzanthracene were buried in the subcutaneous tissue of rats, a finding reported more recently by Vasiliev, Olshevskaja, Raikhlin and Ivanova (1962) and Vasiliev and Guelstein (1963).

Vasiliev *et al.* (1962) conducted a detailed histological and histochemical study on the alterations induced in the connective tissue of rats by 7,12-dimethylbenzanthracene. He demonstrated an inhibition of the formation of granulation tissue around a paraffin pellet containing 2 mg. of the hydrocarbon and a depression of fibroblastic differentiation with large quantities of fibrinoid material present around the capsule. Later investigations using 1 mg., 0·1 mg. and 0·01 mg. of DMBA revealed that all three doses inhibited connective tissue proliferation and depressed fibroblastic differentiation in the first weeks after administration. Isolated disorientated fibroblasts were seen in the tissues at 15–20 days and metachromatically staining acid mucopolysaccharides were demonstrated together with strongly PAS positive fibrinoid.

An analogous inhibition in the proliferation of reparative connective tissue can occur after the application of X-irradiation to a wound. Irradiation 28 hours after surgical infliction of a wound in rats resulted in a reduction of the mesenchymal cell proliferation by 53% when measured on the fifth day following injury (Grillo, 1963). In this respect carcinogens may be said to exercise a local radiomimetic effect, additional to those observed on systemic administration (Boyland, 1957).

A similarity of action to that found in subcutaneous tissue has been observed in the skin, where painting of hydrocarbons in several species has produced a parallel delay in the repair mechanisms of the epidermis. Howes (1946) reported that application of methylcholanthrene to the epidermis of rats resulted in an initially destructive lesion. Repair was inhibited, and after 7–10 days epithelial cells showed a slower rate of division than those of controls. Regenerated collagen fibrils in the dermis were sparse and did not differentiate. Cramer and Stowell (1941) found that carcinogens applied to mouse skin did not induce a proliferation

---

EXPLANATION OF PLATES

FIG. 1.—Structural formulae of N-methyl-N-nitrosourea, N-nitroquinoline-N-oxide, propane sultone and butyrylethyleneimine.

FIG. 2.—Fibrinoid degeneration of collagen (fd) staining red-brown, and fibrin deposit (f) staining blue at the site of 4 injections of methylnitrosourea in rats. P.T.A.H. × 150.

FIG. 3.—Enlarged hyperchromatic fibroblasts (fb) at the site of four injections of propane sultone in rats. H. and E. × 210.

FIG. 4.—Complete absence of granulation tissue formation or reparative processes operative in the subcutaneous site after five injections of nitroquinoline-N-oxide into rats. pc = panniculus carnosus. dm = deep muscle layer. H. and E. × 50.

FIG. 5.—Large hyperchromatic fibroblast from injection site of rat given seven injections of methylintrosourea. Note enlarged nucleolus. H. and E. × 1,400.

FIG. 6.—Electron micrograph of fibroblast from the site of seven injections of methylnitrosourea in rats, showing segregation of nucleolar components. Pb citrate/uranyl acetate. × 12,500.

FIG. 7a.—Electron micrograph of fibroblast from the subcutaneous site of four injections of N-nitroquinoline-N-oxide in rats. The nucleus appears dense, and the nucleolus is prominent. Pb citrate/uranyl acetate. × 10,500.

FIG. 7b.—Higher magnification of nucleolus from 7a, demonstrating segregation of nucleolar elements. × 27,000.

FIG. 8.—Absence of reparative response after 10 injections of methylnitrosourea subcutaneously into rats. H. and E. × 54.

FIG. 9.—Necrosis (n) of fatty tissue and extensive fibrin deposit (f) around a subcutaneous cystic space after five injections of nitroquinoline-N-oxide in guinea pig. H. and E. × 45.

1

2

Hooson, Grasso Gangolli

3

4

5

6

7

8

9

Hooson, Grasso and Gangolli of epithelial cells but injured them and inhibited their mitotic activity for several days. Sharashidze and Bulusashvili (1966) found that a local depression of cell division and proliferation occurred after painting the skin of mice and hamsters with dimethylbenzanthracene and methylcholanthrene. Topical application of carcinogens affects not only epithelium but also the sub-epithelial tissues in rats, mice, rabbits and guinea-pigs. Initially collagen undergoes degeneration but reconstitution of the collagen is considerably delayed after application of a variety of the carcinogenic hydrocarbons topically (Maltoni and Zajdela, 1963).

Other workers compared the wave of mitosis induced in the epithelium of mice by the application of irritants or carcinogens. Elgjo (1968) reported that the peak of mitotic activity after the topical application of 3,4-benzopyrene occurred at about 4 days, whereas with irritants, the peak was at 2 days; Evensen (1961) concluded that similarly applied methylcholanthrene interferes with the synthesis of DNA and the mitotic process immediately after application.

Other examples of growth inhibition by carcinogens illustrate that the phenomenon is not limited to the skin or to the granulation tissue that develops at the site of injury. Laws (1959) demonstrated inhibition of mitotic activity by the administration of fluorenylacetamide in the regenerating liver of rats after hepatectomy. Similarly a single intravenous dose of 7,12-DMBA caused suppression of growth of regenerating liver in partially hepatectomised rats, as shown by a decrease in the mitotic index, inhibition of increase of cell number and total amount of DNA (Marquardt and Philips, 1970). A single intravenous injection of methylazoxymethanol acetate was followed by inhibition of DNA synthesis in rat liver, small intestine and kidney, organs where this compound exerts its carcinogenic effect (Zedeck, Sternberg, Poynter and McGowan, 1970). Oral administration of aromatic mustards, aminostilbenes and carcinogenic hydrocarbons retards the growth rate of rats and mice (Haddow, 1951).

Moreover, carcinogenic hydrocarbons inhibit the proliferation of fibroblasts *in vitro* whilst chemically related but non-carcinogenic hydrocarbons do not (Vasiliev and Guelstein, 1963). Similar inhibition of cell division after contact with carcinogens has been demonstrated using protozoa. Ord (1965, 1968) exposed cultures of *A. proteus* to methylnitrosourethane and found that division was delayed from the normal 2–3 day interval for periods of up to 8 weeks. Amoebae 2–3 times normal size were produced.

Enlargement of cells and production of cytological abnormalities appears to be a feature common to various carcinogenic agents.

Pullinger (1941) observed an increase in the size of both cytoplasm and nuclei in epidermal cells 24 hours after treatment with 0·1% methylcholanthrene in acetone. Nuclear distortion and cytoplasmic vacuolation were also observed. Afzelius and Schoental (1967) reported an increase in the size of parenchymal liver cells 2–3 weeks after a single intragastric dose of carcinogenic pyrrolizidine alkaloids. Ultrastructural and autoradiographical studies supported the view that these enlarged hepatocytes were not degenerate, but that their large size could be interpreted as a failure of such actively metabolising cells to undergo division.

The ultrastructural changes found in the present study of the enlarged fibroblasts at the site of MNU and NQO injections are not readily interpretable.

It is known that the nucleolus is the site of ribosomal RNA synthesis (Perry, 1963; Caspersson, Farber, Folley and Killander, 1963). Administration of many hepatocarcinogens leads to some form of nucleolar rearrangement (Svoboda and Higginson, 1968). It has been suggested that a change in nucleolar morphology represents the inhibition of DNA directed RNA synthesis. However, the specificity of this morphological change after carcinogen treatment must be considered doubtful, since Actinomycin D, a non hepatocarcinogen, has also been shown to produce dissociation of the nucleolar components of hepatocytes (Goldblatt, Sullivan and Farber, 1969) and inhibit RNA synthesis (Reich, 1963). The significance, if any, of such morphological and biochemical changes in the genesis of neoplasia remain to be elucidated.

The appearance of fibrinoid in several animals in the present experiments seems yet another indication of the destructive effect of carcinogens on tissue constituents. Chemical and biochemical studies have shown that carcinogens including the compounds studied by us are capable of interacting and denaturing a variety of intracellular constituents such as nucleic acids and proteins (Rees and Varcoe, 1967; Poirier, Miller, Miller and Sato, 1967; Brookes and Lawley, 1964; Harvey and Halonen, 1968). The fibrinoid is probably the morphological manifestation of the ability of carcinogens to degrade protein, in this case collagen, that was present at the injection site. Alteration of tinctorial properties is a manifestation of this degradation.

### CONCLUSION

The results presented in this work, together with previous observations, tend to indicate that carcinogens suppress cell multiplication and induce cytomegaly. In the subcutaneous tissue these effects are accompanied by a destruction of collagen. The presence of these features in the tissue changes produced by a variety of carcinogenic compounds differing widely in chemical structure would suggest that there might be some connection between pathological processes of this nature and neoplasia resulting from the interaction between a carcinogen and intracellular receptor sites involved in the regulation of cell growth.

The pathological changes in the subcutaneous tissue produced by carcinogens differ widely from those produced by surface active, acidic, hypertonic or amphipathic solutions or solutions of high $Ca^{2+}$ concentration. In these instances the reaction was proliferative in nature and collagen deposition, rather than destruction, was a prominent feature of the pathological changes. These differences reinforce our earlier conclusion that solutions possessing the physical properties mentioned induce the evolution of local malignancy by a mechanism which appears to be different from that of chemical carcinogens.

### REFERENCES

Afzelius, B. A. and Schoental, R.—(1967) *J. Ultrastruct. Res.*, **20**, 328.

Boyland, E.—(1957) *Endeavour*, **11**, 87.—(1958) *Br. med. Bull.*, **14**, 93.

Brookes, P. and Lawley, P. D.—(1964) *Nature, Lond.*, **202**, 781.

Cappellato, M.—(1942) *Tumori*, **16**, 38.

Caspersson, T., Farber, S., Foley, G. E. and Killander, D.—(1963) *Expl. Cell Res.*, **32**, 529.

Cater, D. B.—(1961) *Br. J. Cancer*, **14**, 607.

Cramer, W. and Stowell, R. W.—(1941) *Cancer Res.*, **1**, 849.

Druckrey, H., Kruse, H. and Preussmann, R.—(1968) *Naturwissenschaften*, **55**, 449.

Druckrey, H., Preussmann, R., Ivankovic, S. and Schmahl, D.—(1967) *Z. Krebsforsch.*, **69**, 103.

DRUCKREY, H., STEINHOFF, D., PREUSSMANN, R. AND IVANKOVIC, S.—(1963) *Naturwissenschaften*, **50**, 735.
ELGJO, K.—(1968) *Acta path. microbiol. scand.*, **73**, 183.
EVENSEN, A.—(1961) *Acta path. microbiol. scand.*, Suppl. 148, 43.
FUJINO, H., CHINO, T. AND IMAI, T.—(1965) *J. natn. Cancer Inst.*, **35**, 907.
GANGOLLI, S. D., GRASSO, P. AND GOLBERG, L.—(1967) *Fd. Cosmet. Toxic.*, **5**, 601.
GOLDBLATT, P. J., SULLIVAN, R. J. AND FARBER, E.—(1969) *Cancer Res.*, **29**, 124.
GRASSO, P., GANGOLLI, S. D., GOLBERG, L. AND HOOSON, JEAN—(1971) *Fd Cosmet. Toxic.*, **9**, in press.
GRASSO, P., GANGOLLI, S. D. AND HOOSON, JEAN.—(1969) *Br. J. Cancer*, **23**, 787.
GRASSO, P. AND GOLBERG, L.—(1966) *Fd Cosmet. Toxic.*, **4**, 297.
GRILLO, H. C.—(1963) *Ann. Surg.*, **157**, 453.
HADDOW, A.—(1951) *Proc. R. Soc. Med.*, **44**, 263.
HARVEY, R. G. AND HOLONEN, M.—(1968) *Cancer Res.*, **28**, 2183.
HOWES, E. L.—(1946) *Cancer Res.*, **6**, 298.
JASMIN, G.—(1961) *Revue can. Biol.*, **20**, 701.
LAWS, J. O.—(1959) *Br. J. Cancer*, **13**, 669.
LUSKY, L. M. AND NELSON, A. A.—(1957) *Fedn Proc. Fedn Am. Socs exp. Biol.*, **16**, 318.
MALTONI, C. AND ZAJDELA, F.—(1963) *Acta Un. int. Cancr.*, **19**, 584.
MARQUARDT, H. AND PHILIPS, F. S.—(1970) *Cancer Res.*, **30**, 2000.
MORI, K.—(1962) *Gann*, **53**, 303.
NAKAHARA, W. AND FUKUOKA, F.—(1969) *Gann*, **50**, 1.
ORD, M. J.—(1965) *Nature, Lond.*, **206**, 413.—(1968) *Expl Cell Res.*, **53**, 73.
ORR, J. W.—(1939) *J. Path. Bact.*, **49**, 157.
PARISH, D. J. AND SEARLE, C. C.—(1966*a*) *Br. J. Cancer*, **20**, 200.—(1966*b*) *Br. J. Cancer*, **20**, 206.
PEARSE, A. G. E.—(1968) 'Histochemistry: theoretical and applied'. 3rd Edition. London (Churchill), p. 233.
PERRY, R. P.—(1963) *Expl Cell Res.*, **29**, 400.
POIRIER, L. A., MILLER, J. A., MILLER, E. C. AND SATO, K.—(1967) *Cancer Res.*, **27**, 1600.
PULLINGER, B. D.—(1940) *J. Path. Bact.*, **50**, 463.
REES, K. R. AND VARCOE, J. S.—(1967) *Br. J. Cancer*, **21**, 174.
REICH, E.—(1963) *Cancer Res.*, **23**, 1428.
RONDONI, P.—(1937) *Z. Krebsforsch.*, **47**, 59.
SCHABAD, L. M.—(1935) *Z. Krebsforsch.*, **42**, 295.
SCHREIBER, D., JANISH, W., WARZOK, R. AND TAUSCH, H.—(1969) *Z. ges. exp. Med.*, **15**, 76.
SHARASHIDZE, L. K. AND BULUSASHVILI, R. B.—(1966) *Folia Histochem. Cytochem.* **4**, 237.
SVOBODA, D. AND HIGGINSON, J.—(1968) *Cancer Res.*, **28**, 1703.
TAKIZAWA, N.—(1940) *Gann.*, **34**, 1.
VASILIEV, J. M. AND GUELSTEIN, V. I.—(1963) *J. natn. Cancer Inst.*, **31**, 1123.
VASILIEV, J. M., OLSHEVSKAJA, L. C., RAIKHLIN, N. T. AND IVANOVA, O. J.—(1962) *J. natn. Cancer Inst.*, **28**, 515.
WALPOLE, A. L. (1961) ' Observations upon the Induction of Subcutaneous Sarcomata in Rats '. Proceedings of an International Conference held at the University of Perugia, June 26–30 1961, p. 83. Reprinted from the Morphological Precursors of Cancer, a publication of the Division of Cancer Research, University of Perugia, Italy.
WOLBACH, S. B.—(1936) *Archs Path.*, **22**, 279.—(1937) *Am. J. Path.*, **13**, 662.
ZEDECK, M. S., STERNBERG, S. S., POYNTER, R. W. AND MCGOWAN, J.—(1970) *Cancer Res.*, **30**, 801.